# Using a cash transfer plus SMS nudge package to improve the wellbeing among caregivers of adolescents living with HIV during the COVID-19 epidemic in South Africa: A pilot randomised controlled trial

**DOI:** 10.1371/journal.pgph.0003799

**Published:** 2025-05-16

**Authors:** Stanley Carries, Zibuyisile Mkhwanazi, Nokwanda Sithole, Lovemore Sigwadhi, Mosa Moshabela, Makandwe Nyirenda, Jane Goudge, Eugene Lee Davids, Darshini Govindasamy

**Affiliations:** 1 Health Systems Research Unit, South African Medical Research Council, Durban, South Africa; 2 School of Nursing and Public Health, University of KwaZulu-Natal, Durban, South Africa; 3 Burden of Disease Unit, South African Medical Research Council, Cape Town, South Africa; 4 Centre for Health Policy, University of the Witwatersrand, Johannesburg, South Africa; 5 Department of Psychology, Faculty of Humanities, University of Pretoria, Pretoria, South Africa; University of the Witwatersrand Johannesburg Faculty of Health Sciences, SOUTH AFRICA

## Abstract

Caregivers of adolescents living with HIV encounter multiple economic and psycho-social challenges which impair their wellbeing and provision of optimal care. Cash transfers combined with short message service (SMS) nudges may address the financial and mental barriers to caregiver wellbeing in sub-Saharan Africa. We examined the preliminary effectiveness and feasibility outcomes of this multipronged approach for improving caregiver wellbeing. We piloted the Caregiver Wellbeing intervention in the eThekwini municipality, KwaZulu-Natal, South Africa. Participants were randomly assigned to one of the following groups: (i) the intervention arm (n = 50) received three cash payments (of ZAR 350, approximately 21 USD), coupled with behaviourally-informed mobile SMS nudges over a 3-month period; (ii) the control arm (n = 50) received a standard SMS encouraging linkage to health services. The primary outcome was change in psychological wellbeing at four-months follow-up. Secondary outcomes were changes in depressive symptoms and caregiver burden scores, recruitment pace, retention, uptake, acceptability and costs. Trial Registraion Number: PACTR202203585402090. The n = 100 caregivers (mean age = 42.3 years, 87% female) enrolled at baseline were recruited within six weeks. Compared to controls, there was a non-significant increase in psychological wellbeing (β = 3.14, *p = *0.319). There was a 1.32 unit (*p* = 0.085) decrease in depressive symptoms and a reduction in caregiver burden (β = -1.28, *p* = 0.020) in the intervention arm. Participant retention was 85%, with high intervention uptake (95%). Caregivers expressed appreciation for the intervention as the cash component allowed them to fulfil their carer responsibilities and the SMS brought a sense of belonging and self-acceptance. Total societal cost of the intervention was US$13,549, and the incremental cost per increase in wellbeing score was US$1,080. Results suggest a cash transfer plus SMS nudge package, whilst feasible and acceptable, may require longer duration and an economic empowerment component to enhance caregiver wellbeing as part of post-pandemic recovery efforts.

## Introduction

Sub-Saharan Africa (SSA) is home to an estimated 85% of the 1.65 million adolescents living with HIV (ALHIV) [1]. The lack of comprehensive health services for ALHIV in SSA means that their informal caregivers are often left to fill this critical care gap with minimal financial and psycho-social support [2] and high personal costs [3]. This ultimately threatens caregiver wellbeing, which is primarily derived from fulfilling social roles and one’s sense of belonging in this context [4,5]. Optimal caregiver wellbeing is associated with health benefits for ALHIV [6,7]. Prioritising the wellbeing of those in the unpaid care economy was a key COVID-19 recovery strategy for progress towards achieving Sustainable Development Goal 3 [8]. Hence it is vital to identify effective and feasible interventions that promote caregiver wellbeing to inform post-pandemic recovery policies in the region.

The economic hardships and social isolation linked to the COVID-19 pandemic may have further compromised wellbeing, increasing mental health problems among caregivers in SSA [9,10]. Behavioural economics-informed interventions in the field of HIV and public health provide insight for the development of low-cost solutions that can address the poverty and psycho-social barriers to caregiver wellbeing [11]. The Caregiver Wellbeing (CWEL) pilot trial used a multipronged approach that includes a cash transfer plus a Short Message Service (SMS) nudge [12]. Informed by current literature, the use of cash transfers or financial incentives have been used for the improvement of health and wellbeing among vulnerable groups during and post-pandemic [13]. Cash transfers have been found to be effective in improving HIV care cascade outcomes (i.e., prevention, testing, linkage, retention) [14] as well as mental health and wellbeing among adults in low-and-middle income countries [15] Evidence on cash transfers for improving wellbeing among caregivers of ALHIV during the COVID-19 pandemic is scarce. Integrating a behavioural intervention that encourages caregivers to adopt positive mental health coping strategies, such as a nudge-based intervention, could have favourable wellbeing effects.

Nudges alter the way options are presented and are designed to influence decision-making without restricting individual’s choices [16]. Studies using nudges in the form of messages that leverage behavioural science principles such as people’s general desire to avoid physical or emotional losses (“loss aversion”) have reported reductions in missed clinic appointments among people living with HIV in South Africa [17] and mental health conditions in the United States of America [18]. Specifically, SMSs that reminded residents in Japan of the benefits of adherence to COVID-19 preventative measures for protection of their families health (“altruism”) was associated with increased COVID-19 vaccine uptake [19]. And finally, online messages highlighting the importance of prioritising ones mental health needs first in order to help others (“framing”) was associated higher uptake of mental health services among students in the Unites States of America [20]. Nudge-based mobile messages that draw on similar principles to promote mental health could be a low-cost and effective solution for enhancing caregiver wellbeing.

To date, there have been no trials on economic interventions for improving caregiver wellbeing during COVID-19 in SSA. Moreover, the effect of combining cash transfers with nudge-based SMS, is unknown. The aim of this study was to conduct a pilot randomised controlled trial (RCT) to evaluate the preliminary effectiveness and feasibility of an economic package (i.e., cash transfer plus nudge SMS package) for improving wellbeing among caregivers of ALHIV. We hypothesised that the intervention, as compared with usual care, would have higher levels of reported psychological wellbeing and lower levels of depressive symptoms and caregiver burden. In addition, we hypothesised that the intervention would be feasible to implement based on recruitment pace, retention, uptake, acceptability, and costs.

## Methods

The methods of the CWEL trial have previously been published elsewhere [12]. A brief overview is provided here. The Extended Consolidated Standards of Reporting Trials guidelines were followed to report the results of this trial [21].

### Ethics statement

Written informed consent was obtained from all participants. Ethics approval was obtained from the South African Medical Research Council’s Human Research Ethics Committee (EC036–8/2021) and permission to conduct this research within the healthcare facilities was obtained from the South African National Department of Health (KZ_202112_005). Participants received cash (R50, ~ 3 USD) per interview completed to reimburse them for their time. As baseline interviews were conducted face-to-face, participants also received a lunch pack and transport money to the value of R100 (~ 6.10 USD). The trial has been registered in the Pan African Clinical Trials Registry (PACTR) PACTR202203585402090; URL: https://pactr.samrc.ac.za/.

### Study design, setting and eligibility

The CWEL trial was a two-arm pilot RCT conducted between November 2021 and March 2022. Caregivers were recruited between 01 November and 15 December 2021 from one public-sector out-patient adolescent HIV clinic, located in the south of the eThekwini municipality, KwaZulu-Natal, South Africa. Information sessions were conducted with caregivers during clinic waiting periods. Trial staff arranged screening appointments at our study office with caregivers who expressed interest in the study. Caregivers were eligible for inclusion if they were the primary caregiver (biological or non-biological) of an ALHIV (aged 10–19 years), were aged 18 years and above, and had their own mobile phone. Caregivers who provided written informed consent in English or isiZulu were enrolled in the trial. Once enrolled, caregivers completed a baseline questionnaire and were randomised to receive the intervention or control over three months. At month four, they were followed up for endline interviews (15 March-30 April 2022) (S1 Fig).

### Intervention arm

The intervention was co-developed with our caregiver advisory board members (CAB) (n = 16). These board members were caregivers of ALHIV accessing care at the study clinic and were not participants in the trial. Drawing on elements of human-centred design thinking [22], we hosted three participatory workshops with the CAB to understand how they experience wellbeing, their needs and barriers to wellbeing. We used these workshops to obtain feedback on the initial intervention prototype and co-write the script for the nudge SMS. We refined the intervention based on workshop feedback and presented it to the CAB and other experts in the field prior to finalisation.

The CWEL intervention group received a monthly cash transfer and nudge messages over a three-month period. The cash transfer was unconditional and amounted to R350 ZAR ($21 USD) per month. This amount was informed and equivalent to the South African government’s monthly COVID-19 Social Relief of Distress grant that was being administered to vulnerable households [23]. This cash transfer was delivered via a local banking service. Participants could only withdraw the money from the bank’s automated teller machine (ATM) located in public spaces. They had to retrieve the money within 30 days using a unique bank password and payment code that was sent to their mobile phones via SMS for each transfer at the start of each month. Thereafter, fieldstaff texted the standard SMS nudge messages (in English and isiZulu) to the participant’s mobile phone. Each message drew on one of the three behavioural economic principles (loss aversion; aspiration framing; altruism) and emphasised aspects that are important to achieving social wellbeing in this context and what the CAB expressed as key to their wellbeing (e.g., fulfilment of role-relationships, positive mental health coping, aspirations) [24] (S1 Table).

### Control arm

The control arm received a once-off SMS encouraging access to government-run healthcare facilities as per the standard message sent by the South African Department of Health to public-sector patients. The control arm did not receive any component of the intervention (i.e., cash transfer or nudge messages).

### Baseline and endline trial assessments and data collection

At enrolment, all participants completed a baseline questionnaire. These questionnaires were pre-programmed on REDCap in English or isiZulu and were administered face-to-face by trained field staff using a Tablet, with strict adherence to COVID-19 protective measures. The baseline questionnaire collected data on socio-demographics, household income, expenses, costs related to accessing care, food insecurity, psychological wellbeing (Mental Health Continuum Short Form (MHC-SF)) [25], subjective wellbeing (Carer Quality of Life (CarerQoL)- happiness score) [26], depressive symptoms (Centre for Epidemiological Studies Depression Scale-10 items (CES-D-10)) [27], caregiver burden (CarerQoL 7D scale)) [26]. The MHC-SF is a short 14-item scale, examining subjective, psychological and social wellbeing (e.g., “*During the past month, how often did you feel like you belonged in your community?”)*. This scale has exhibited moderate internal consistency (Cronbach’s alpha = 0.74) and good criterion validity (0.3-0.52) with other scales measuring similar constructs among a South African sample [25] and was formally translated to the local language (isiZulu) and pilot tested with a group of caregivers for this trial [12]. The CarerQol-7D scale [26] is composed of two parts: (ii) the visual analogue scales (VAS) which measures subjective wellbeing (happiness) on a scale from 0 to 10; ii) the CarerQol-7D, which describes the care situation in terms of the negative and positive effects of caregiving (e.g., “I have no/some/a lot of financial problems because of my care tasks?”). In a recent study, this scale demonstrated moderate internal consistency (Cronbach’s alpha = 0.65) and strong convergent validity (r = 0.90) among informal Iranian caregivers [28]. The CES-D-10 scale was used to identify symptoms suggestive of probable depression. It consists of 10 items that assess how an individual felt in the past week. Sample items include; “I was bothered by things that don’t usually bother me”, “I had trouble keeping my mind on what I was doing”. The levels of severity for each of the items ranged from: rarely or none of the time (score 0), some or little of the time (score 1), occasionally or moderate amount of time (score 2), and almost or all of the time (score 3). The depressive symptom score is constructed as the sum over all responses to all ten items. Therefore, the CES-D-10 cumulative score ranges from 0 to 30, with a higher score reflecting a higher depressive symptom risk profile. A cut-off score of 12 or higher is deemed optimal to correctly classify individuals as having probable depressive symptoms for a South African sample [27]. The CES-D-10 has demonstrated acceptable internal consistency across various language groups in South Africa (Cronbach’s alpha = 0.69-0.89), and concurrent validity when compared to other commonly used depressive symptom measures [27].

Clinical data (e.g., HIV viral load), was extracted from HIV clinic records. Once randomised, n = 8 caregivers in each arm were purposively sampled based on age, gender and HIV-status for baseline semi-structured in-depth interviews (IDIs) that probed key aspects of the baseline questionnaire (i.e., stigma experiences, mental health coping, caregiving challenges). These IDIs were conducted by a trained fieldstaff member in the participant’s preferred language using a topic guide. Once the intervention and control were delivered, all participants were telephonically followed up for their endline interview at month four which collected similar data to that in the baseline questionnaire. Participants who completed baseline IDIs were also followed up face-to-face for endline IDIs once their trial endline survey was complete. Endline IDIs explored similar topics covered in the baseline topic guide as well as their feedback on the intervention, control and participation in the trial. All IDIs were audio-recorded, transcribed and translated.

At the end of each month, data were collected from trial monitoring documents on the number recruited per week, safety concerns reported by participants, adverse events, referrals reported, date of intervention and control delivery, and number of intervention participants that retrieved their cash transfer and nudge SMS.

Cost data were also collected alongside the trial using the micro-costing approach described in the Global Health Cost Consortium guidelines [29]. We collected fixed (i.e., building, vehicle, furniture and equipment, intervention development) and variable costs (personnel, supplies, utilities, caregiver-side costs). All research-related costs were excluded. We obtained cost estimates from the trial’s financial documents, rosters and logbooks (S2 Table). Overheads and other shared costs were allocated based on days spent on set-up and delivery. To gauge personnel time, we undertook a combination of direct observation and drew on staff timesheets.

### Outcome measures

There were two primary outcomes: (i) psychological wellbeing- change in the mean MHC -SF score at baseline and endline between the two arms; (ii) subjective wellbeing- change in the mean Carer QoL happiness score at baseline and endline between the two arms. See S3 Table for further details on the scale items and scoring. Secondary outcomes included changes in depressive symptoms, caregiver burden scores and feasibility. We assessed feasibility by assessing five factors that can affect further evaluations (recruitment rate, retention rate) and intervention implementation (uptake, participant acceptability, and average cost per increase in psychological wellbeing score).

### Sample size, randomisation

Given the repeated measures study design, our sample size was determined by fitting a repeated-measures Analysis of Variance (ANOVA) using the univariate *F* test. The sample size of n = 100 provided us with 80% power to detect a mean difference in subjective wellbeing scores in the range of δ = 0.6 to δ = 0.5 at a 5% significance level. We postulated that the variance between means would be in the range of 2.3 to 2.6, with a correlation between repeated measurements at baseline versus endline of 0.7 (S4 Table) [30]. We consider the treatment effect size meaningful for the short trial duration given that people derive wellbeing from multiple social networks in this context making it more complex to change in the short-run [5].

We conducted randomisation at the individual level by allocating participants to one of two arms, intervention or control arm (1:1). A statistician (LS) masked to baseline information used block randomisation with random block sizes of two, four, eight, and twelve, assigning participants to one of two trials arms using STATA (Stata Corp, College Station, TX, V 16). Stratification factors were not included in the randomisation process.

### Analysis

We compared baseline data (socio-demographic, health and wellbeing status) by arm using Chi-square tests for categorical data and Kruskal-Wallis H tests for continuous variables. No adjustment for multiple comparisons was applied as we only had 2 arms (intervention versus control). Pre-post differences in outcome between arms were assessed descriptively (change in mean score or percentage at baseline versus enrolment) and using a linear regression model for continuous outcomes to obtain beta coefficients, 95% CIs, and *p* values. As we examined changes in continuous outcomes at two time-points we used the Analysis of Covariance (ANCOVA) method, adjusting for baseline covariates (age and sex) and using an intention-to-treat approach [31]. Additional robustness checks were done using linear and log-binomial models. Statistical analyses were performed using STATA (Stata Corp, College Station, TX, V 18).

We used the framework method for the analysis of the qualitative data [32]. This entailed, transcribing and translating audio files, reviewing the transcripts, reviewing debriefing notes from the field team, and discussing content with the field team. A research assistant coded initial transcripts using NVivo 12. Codes were grouped into categories (e.g., timing of the intervention, intervention components, intervention receipt, SMS message content) and then grouped into themes based on the domains from the Theoretical Framework of Acceptability (TFA) [33]: affective attitude (*how individuals feel about taking part in an intervention*), burden (*the amount of effort required to engage with an intervention*), perceived effectiveness (*whether individuals perceive an intervention as likely to achieve its purpose*), ethicality (*the extent to which an intervention fits with individuals’ values*), intervention coherence (*whether individuals understand an intervention and how it works*), opportunity costs (*what is given up, such as time, to take part in an intervention*) and self-efficacy (*how confident individuals are doing the intervention*). Once the framework was finalised, we applied this to the remaining transcripts via coding and updating the file with any new codes and categories based on the data. We then extracted key quotes from transcripts that exemplified a category or theme and interpreted the data jointly with the field and investigator team.

The cost analysis was conducted using a provider and societal perspective, using a combination of bottom-up and top-down costing approaches. We conducted a descriptive analysis of total economic cost for key cost categories (fixed, variable) by arm, and estimated the total provider and total societal cost. The average cost per person with an increase in psychological wellbeing was presented. For the cost-effectiveness analysis, we assessed incremental cost effectiveness ratios (ICERs), which is a summary measure of the expected incremental costs to the incremental benefits of the intervention compared with the standard of care. The ICERs were calculated by dividing the difference in the average cost per participant in each arm by the difference in the proportion of participants with an increase in wellbeing score by arm. As South Africa does not have a cost-effectiveness threshold, we used the widely used Gross-Domestic Product (GDP) threshold which deems an intervention cost-effective if its below 1–3 × the country’s GDP per capita [34]. All costs were expressed in 2022 $US at an exchange rate of $1USD = R16.37 ZAR.

## Results

### Study flow

**Fig 1:**
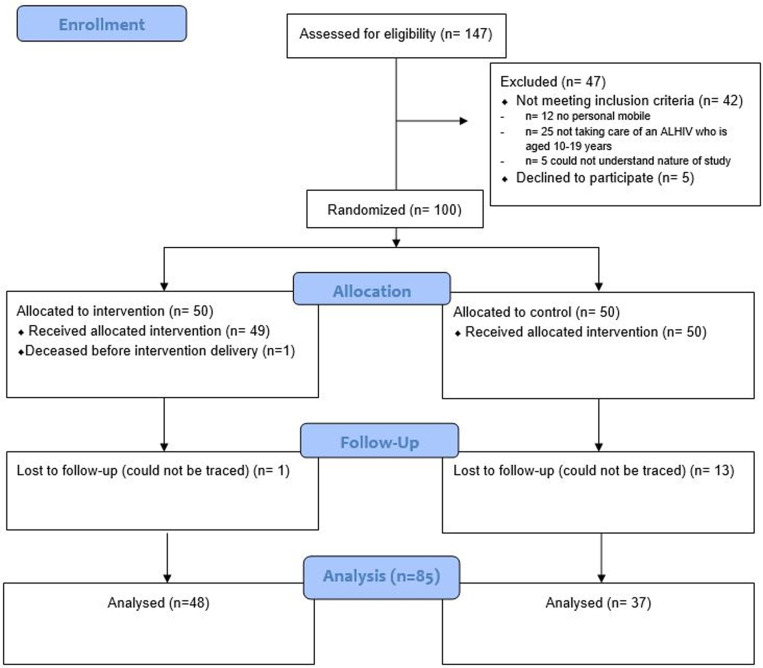
Participant Flow.

During recruitment, n = 147 caregivers of ALHIV were approached and assessed for eligibility of whom n = 5 refused to participate and n = 42 were ineligible due to the following reasons (n = 12 did not have a mobile phone, n = 25 were not taking care of an ALHIV aged 10–19 years, n = 5 could not understand the study procedures). The remaining n = 100 were enrolled, completed baseline assessments at month 0, were randomised to an arm (n = 50 intervention, n = 50 control), and n = 8 per arm were sampled for baseline IDIs (Fig 1). The intervention and control delivery occurred during month 1–3, with 1 participant reported deceased in the intervention arm prior to intervention delivery. At follow-up, n = 14 were deemed lost to follow-up as they could not be traced (n = 1 intervention, n = 13 control). Endline assessments were conducted with n = 85 participants at month four (n = 48 intervention, n = 37 control). Additionally, clinical data were analysed for n = 69 participants (n = 38 intervention, n = 31 control) who had laboratory data available at baseline and endline.

### Baseline characteristics

Socio-demographics of participants were balanced across arms at baseline. The mean age in our sample was 42.3 years and most caregivers were female (87%). Nearly 60% were unemployed and more than half were married. Just over two-thirds of caregivers reported receiving a social grant and caring for an ALHIV aged 13 years. Half of the participants resided in households experiencing severe food insecurity. The majority of caregivers were living with HIV (93%) and had suppressed HIV-viral loads (77%). Most participants were experiencing psychological wellbeing levels that were moderate to languishing (51%) and moderate subjective wellbeing (mean happiness score = 5). More than two-thirds of caregivers in the sample had scores suggestive of depressive symptoms, and this was higher in the intervention (82%) versus the control arm (64%). There was a high prevalence of caregiver burden (59%), with 78% who reported experiencing some form of stigma daily (Table 1).

**Table 1:** Baseline characteristics of sample by trial arm.

	Total (N = 100), N (%)	Intervention (n = 50) N (%)	Control (n = 50) N (%)
***Socio-economic***			
**Age**			
Mean age (SD)	42.3 (11.6)	42.1 (10.6)	42.7 (12.6)
**Age categories**			
*< 60 years*	91 (91.0)	47 (94.0)	44 (88.0)
*≥ 60 years*	9 (9.0)	6 (12.0)	3 (6.0)
**Sex**			
Female	87 (87.0)	45 (90.0)	42 (84.0)
Male	13 (13.0)	5 (10.0)	8 (16.0)
**Employment status**			
Unemployed	59 (59.0)	30 (60.0)	29 (58.0)
Employed	41 (41.0)	20 (40.0)	21 (42.0)
**Relationship status**			
Married	59 (59.0)	30 (60.0)	29 (58.0)
Single	41 (41.0)	20 (40.0)	21 (42.0)
**Recipient of a government social grant/s**			
Yes	67 (67.0)	35 (70.0)	32 (64.0)
No	33 (33.0)	15 (30.0)	18 (36.0)
**Age of ALHIV caring for (years)**			
Median age (IQR)	13 (12-16)	13 (12-17)	13 (12-15)
**Household food insecurity status (FIES)**			
Moderate/None	49 (49.0)	19 (38.0)	30 (60.0)
Severe	51 (51.0)	31 (62.0)	20 (40.0)
***Health and wellbeing***			
**HIV-status**			
HIV-positive	93 (93.0)	43 (86.0)	40 (80.0)
HIV-negative	7 (7.0)	7 (14.0)	10 (20.0)
HIV virological suppression*			
Suppressed	43 (77.0)	23 (72)	20 (83)
Not suppressed	13 (23.0)	9 (28.0)	4 (17.0)
Median CD4 count (cells/µl)**	760 (534-879)	753.5 (534-871)	766.5 (547-902)
**Psychological wellbeing (MHC-SF)**			
**Reported psychological wellbeing states:**			
Flourishing	49 (49.0)	21 (42.0)	28 (56.0)
Languishing^$^/Moderately mentally healthy	51 (51.0)	29 (58.0)	22 (44.0)
**Mean psychological wellbeing score**	46.83 (15.27)	45.82 (15.89)	47.84 (14.70)
**Subjective wellbeing** **(Carer QoL VAS)**			
**Median happiness score (IQR)**	5.0 (4.0-8.0)	5.0 (3.0-7.0)	5.5 (5.0-8.0)
**Depressive symptoms (CESD-10)**			
**Reported depressive symptoms**			
Yes	73 (73.0)	41 (82.0)	32 (64.0)
No	27 (27.0)	9 (18.0)	18 (36.0)
**Mean depressive symptom score (SD)**	14.9 (5.4)	13.8 (5.3)	16.1 (5.3)
**Caregiver burden** **(Carer QoL)**			
**Experiencing caregiver burden**			
Yes	59 (59.0)	31 (62.0)	28 (56.0)
No	41 (41.0)	19 (38.0)	22 (44.0)
Mean caregiver burden score* (SD)	54.1 (19.4)	49.2 (21.4)	59.5 (15.4)
**Experiencing stigma (EDS)**			
Yes	78 (78.0)	39 (78.0)	39 (78.0)
No	22 (22.0)	11 (22.0)	11 (22.0)

• 31% had missing viral loads at baseline, 31% had missing CD4 count data at baseline.

• Virological suppression defined as (based on biomarker HIV-RNA data: ≤ 40 copies/mL = suppressed; > 40 copies/mL = unsuppressed).

• Psychological wellbeing: Summation of the 14-item scale, total score range 0–70. Flourishing: reporting of hedonic or eudaimonic states frequently (almost everyday/everyday). Languishing- reporting of hedonic or eudaimonic signs less frequently (never-almost once a week). Moderately mentally healthy- neither flourishing nor languishing. $ Languishing: As N = 6 participants were at this level (n = 3 intervention, n = 3 control), these participants were grouped with those in the moderately mentally healthy level.

• Subjective wellbeing: 1 item-Total score ranging from 1-10.

• Depressive symptoms: Summation of the 10-item scale, total score range 0–30. Reported symptoms of depressive symptoms defined as CESD-10 score ≥12.

• Caregiver burden: Score generated using Polish tariff. Scores above the median defined as experiencing caregiver burden.

• Abbreviations: Abbreviations: SD = standard deviation; IQR = interquartile range; MHC-SF = Mental Health Continuum Short Form; Carer QoL VAS = Caregiver Quality of Life- Visual Analogue Scale; CESD-10 = Center for Epidemiologic Studies Depression Scale- 10 items; Carer QoL = Caregiver Quality of Life Scale; EDS = Everyday Stigma Scale; FIES = Food Insecurity Experience Scale.

### Feasibility

#### Trial implementation.

Recruitment rate: The trial enrolled n = 100 caregivers within a 7-week period (approximately 14 per week) (Fig 1)

Retention rate**:** Of the n = 100 enrolled in the trial, 85% were retained at endline, with higher retention in the intervention (96%) versus control (74%) arm (Fig 1).

Uptake of intervention**:** In the intervention arm, 86% of the intervention participants received and retrieved all cash transfers within 30 days of delivery and acknowledged receipt of the nudge SMS (Table 2). Of the n = 7 participants that did not receive the full package, most indicated it was due to changes in their mobile number or phone damages at month 2.

**Table 2:** Uptake of the intervention (N = 49).

	n (%)
Received full intervention package	42 (86%)
Month 1	49 (100%)
Month 2	42 (86%)
Month 3	49 (100%)

### Preliminary effectiveness

Primary outcomes were assessed for n = 85 participants at endline, with fewer assessed in the control arm (n = 37, 74%) versus the intervention arm (n = 48, 96%) (Fig 1). There were no statistical differences in key baseline characteristics (e.g.,: age, gender, HIV status) of those participants retained versus those lost to follow-up or deceased at endline in each arm (S5 Table).

**Fig 2:**
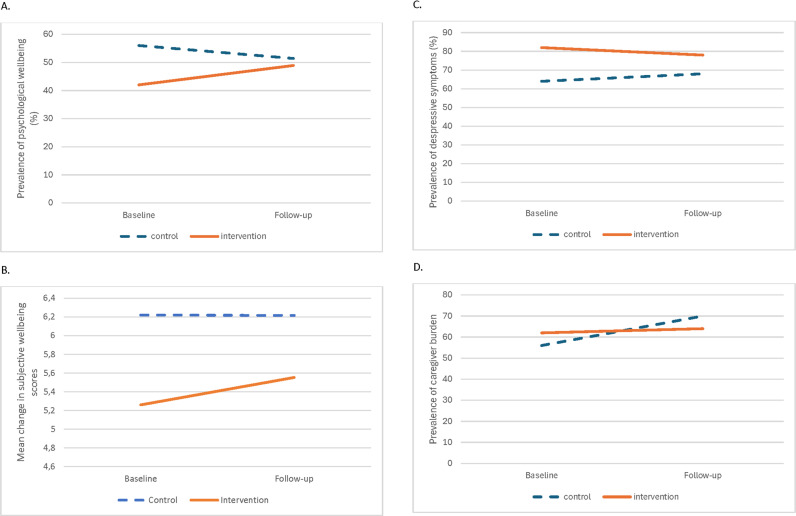
Changes in observed scores of A) psychological wellbeing (Fig 2A), B) subjective wellbeing (Fig 2B), C) depressive symptoms (Fig 2C), and D) caregiver burden over four months among caregivers by treatment arm (Fig 2D).

Despite a lack of statistical significance, psychological wellbeing increased by 6.9 percentage points in the intervention arm and decreased by 4.6 percentage points in the control arm at baseline versus endline (Fig 2). Similarly, there was a non-significant increase in subjective wellbeing scores by 0.3% in the intervention versus a decrease of 0.04% in the control. Depressive symptoms decreased in the intervention arm (4 percentage points) and increased in the control arm (4 percentage points) at baseline versus endline, yet this trend was not statistically significant. A decrease in caregiver burden was only observed for the intervention arm (2 percentage points) (p > 0.05).

The primary outcome of psychological wellbeing was higher in the intervention versus the control at endline (β = 3.14; 95% CI -3.09 to -9.37, *p* value 0.319), albeit non-significant (Table 3, S6 Table). There was an improvement in subjective wellbeing (β = 1.05; 95% CI: -0.15 to 2.25, *p* value 0.085) and depressive symptom scores (β = -1.32; 95% CI: -3.3 to -0.68, *p* value 0.193) in the intervention versus the control arm at endline. There was a significant decrease in caregiver burden scores (β = -1.28; 95% CI: -2.35 to -0.21, *p* value 0.020) in the intervention versus the control arm at endline. Our results were similar when we used an alternate model specification (S7 Table).

**Table 3:** Changes in continuous outcomes at baseline versus endline (M4) (full sample) assessed using the ANCOVA method.

Outcome	β (95% CI)		Analysis			
Continuous^*^	**Intervention**	**Control**	**Unadjusted** **Mean difference (95% CI)**	**p-value**	**Adjusted**^ **Mean difference** **β (95% CI)**	**p-value**
Primary outcome:						
Psychological wellbeing (MHC- SF)*	1.36 (-2.99-5.71)	-1.27 (-5.36 to 2.82)	3.14 (-3.09 to 9.37)	0.319	0.22 (-4.82 to 5.26)	0.931
Subjective wellbeing (Carer QoL VAS)*	0.51 (-0.39-1.40)	-0.54 (-1.30 to 0.22)	1.05 (-0.15 to 2.25)	0.085	-0.18 (-1.07 to 0.72)	0.698
Secondary outcome:						
Depressive symptoms (CESD-10)	-1.38 (-2.75-0.001)	-0.05 (-1.53 to 1.42)	-1.32 (-3.3 to -0.68)	0.193	0.67 (-1.15 to 2.49)	0.465
Caregiver burden (Carer QoL)	-0.47 (-1.20-0.27)	0.81 (0.03 to 1.60)	-1.28 (-2.35 to -0.21)	0.020	-0.36 (-1.21 to 0.49)	0.399

*= Analysis of Covariance (ANCOVA); ^* = adjusted for baseline values, age and sex at baseline; Abbreviations: MHC = Mental Health Continuum Short Form; Carer QoL VAS = Caregiver Quality of Life- Visual Analogue Scale; CESD-10 = Center for Epidemiologic Studies Depression Scale- 10 items; Carer QoL = Caregiver Quality of Life Scale*

#### Acceptability of the intervention.

**Affective attitude.** Participants described positive attitudes (i.e., feeling happy) upon receipt of the intervention. Many described the messaging content as instilling a sense of self-worth and being a source of support and “encouragement”, and they appreciated that messages were personalised with their names. Some of the excepts shared by participants included:

*“I felt revived in my spirit that even when you know that you have no one in the world but there are people who care for you. That made me very happy.” PID 103, 46-year-old female*

*“Those messages brought comfort and made me feel like a human being and gave me the encouragement I needed”. PID 101, 57-year-old male*

**Burden.** Most participants indicated that they previously utilised the cash transfer system and thus were able to access their money without challenges. However, a few caregivers who had no prior experience with this cash transfer system reported that the instructions for withdrawal were “confusing” and sought assistance from bank staff. One of the participants expressed this when saying:

*“No, it was my first time. When I got the SMS with the withdrawal pin, I was a bit confused, and the bank staff helped me and told me first read the screen of the ATM and enter the 10-digit code.” PID 102, 57-year-old male*

One participant who was living with co-morbidities indicated that when she was unwell and having to go to the bank to withdraw the money placed an added burden on her and would have preferred if the money was delivered to her.

*“It burdens me because sometimes I am not well and I am forced to go to the bank, but maybe if it is possible that you deliver it to me by hand it would be better”* PID 103, 46-year-old female

**Perceived effectiveness.** Several participants indicated that the SMS content “motivated” them and helped them cope with their daily caregiving stressors, one of the participants expressed this by saying:

“Yes, it was encouraging and motivated me to carry on with my life. That’s why I’m keeping the message because it keeps me going.” PID 102, 57-year-old male

Participants generally perceived the cash transfer as an effective solution for reducing household food insecurity during COVID-19, this can be seen in the following remark made by one of participants:

*“I would like for this cash to continue because it is very helpful it gets rid of hunger…it will help a lot of people who are experiencing lack like me during COVID-19.” PID 103, 46-year-old female*

**Ethicality.** Participants also appreciated that the SMS nudge was in their local language (isiZulu) and brief.

*“It was also sent in isiZulu, which is my mother tongue, they were not long.” PID 101, 57-year-old male*

When we probed opinions about the cash transfer amount, most participants stated that they would have appreciated a higher monthly amount, an example of this was when one of the participants provided a suggested monthly amount when saying: “*R800 at a least or a R1000.*” *PID 105, 43-year-old female.*

**Intervention coherence.** There was evidence that participants understood the SMS content as many indicated it raised awareness on “budgeting” and referral services for mental health support.

*“It gave us that hope and also gave us the number to call when feeling depressed.” PID 104; 31-year-old female*

There was also evidence that participants used the money towards fulfilling their caregiving responsibilities as most reported using the cash transfer to purchase essential household food items or pay towards their children’s school transport, uniforms or lunches. One participant echoed this sentiment when she said:

*“Ever since I started receiving the R350 I was able to buy mealie meal, potatoes and so forth. We even ate meat something that we were not used to. The R350 assisted my kids for transportation to school.” PID 105; 43-year-old female*

**Opportunity costs.** Some participants had to borrow money for transport to access the ATM for cash withdrawal. One participant shared the following:

*“The third time withdrawing the cash... I did not have the transport money to go and withdraw but I asked the neighbours for bus fare.” PID 101, 57-year-old male*

**Self-efficacy (how confident individuals are doing the intervention).** Participants also highlighted that the intervention inspired them to start their own small businesses, as can be seen from the following quote:

*“I wish to open a vending table selling detergents that could accumulate money for me.” PID 105; 43-year-old female*

However, none of the intervention participants utilised the mental health service provider listed on the SMS. An example of this can be seen from the following quote from one of the conversations with the participant:

*“I have never tried calling the XX organisation.” PID 103, 46-year-old female*

Most intervention participants indicated that in the months they obtained the economic incentive, they were less dependent on borrowing money informally from local community members.

*“The money helped me skip the loan sharks, skipping the loan sharks is progress for me. I do not want these people getting used to me because they can come in the house and take everything.” PID 107, 47-year-old male*

#### Costs.

Table 4 presents the cost composition by arm. The total societal cost of the intervention arm (US$13,549) was almost double the cost of the control arm (US$6,280). For the intervention arm, variable costs accounted for 62% of the total costs, mainly driven by the supplies (cash transfers). Whereas in the control arm, fixed costs linked to building, furniture and equipment were the main driver. It is important to note that indirect caregiver-side costs were higher in the intervention than the control arm and that was linked to transport costs to the nearest ATM for the cash withdrawal. The average societal cost per participant was higher in the intervention (US$ 271) versus the control (US$ 261) arm. The average incremental provider cost per caregiver with an increase in psychological wellbeing score in the intervention versus the control was US$1,080 (below the threshold of <3 x per capita of South Africa’s 2023 gross domestic product (GDP) of $6,490) (S8 Table).

**Table 4:** Economic cost composition by arm and outcomes.

	Cost in USD ($)	
	Intervention	Control
**FIXED COSTS**		
Building	$2,688	$733
Vehicle	$113	$0
Furniture & equipment	$1,540	$388
Developmental cost		
Manual & database development	$345	$147
Trainings	$467	$250
**Total fixed costs**	**$5,151**	**$1,518**
**VARIABLE COSTS**		
Operational staff	$817	$250
Supplies	$3,488	$147
Utilities	$3,482	$214
Caregiver out-of- pocket payment	$611	$305
**Total variable costs**	**$8,398**	**$916**
**Total cost**	$13,549	$2,434
**Average cost per participant- provider perspective**	$259	$43
**Average cost per participant- societal perspective**	$271	$49
Average cost per participant with an increase in PWB^*^ **score- societal perspective**	$589	$187
Incremental cost-effectiveness ratio (ICER) ** #- provider perspective	$1,080	
Incremental cost-effectiveness ratio (ICER) ** #- societal perspective	$1,100	

*PWB= psychological wellbeing (MHC-SF).

#ICER = Difference in average cost in intervention versus control/ difference in proportion with an increase in psychological wellbeing score in intervention vs control.

## Discussion

Adolescents living with HIV experience multiple challenges that are associated with their HIV status as well as the varied environmental, economic, cultural and social factors [35]. These challenges and factors often result in a significant burden on their caregivers. The financial and mental health burden on caregivers of ALHIV frequently goes unnoticed, particularly in SSA [3]. Economic empowerment and provision of psycho-social support may reduce the financial burden on caregivers of ALHIV, which could yield improved mental health and wellbeing outcomes for caregivers of ALHIV. Therefore, the current pilot RCT aimed to evaluate the preliminary effectiveness and feasibility of a cash transfer plus nudge SMS package for improving wellbeing among caregivers of ALHIV in KwaZulu-Natal, South Africa.

Our trial found no significant improvement in the primary outcome (psychological wellbeing) in the intervention versus control arm. However, at follow-up, results favoured the CWEL intervention arm, namely the provision of a cash transfer plus the nudge SMS package, which showed improvements in wellbeing and mental health outcomes based on pre-post score differences. When compared to the control arm, the intervention was associated with notable improvements in psychological wellbeing (β = 3.14, p = 0.319), subjective wellbeing (β = 1.05, p = 0.085) and depressive symptoms (β-1.32, p = 0.193), albeit non-significant. Our findings are aligned with a pre COVID-19 Zambian study which found that a cash transfer programme combined with psycho-social support was associated with improvements in caregiver mental health and wellbeing [36]. Whilst not measured in this trial, it is likely that increased caregiver wellbeing levels in the intervention arm may have been associated with improved HIV treatment outcomes among ALHIV. This could have been due to the fact that carers now had the resources to alleviate some of the financial stresses linked to caregiving, and the messages may have made them feel more hopeful resulting in positive attitudinal changes towards their ALHIV and provision of more emotional support with regards to disclosure, treatment adherence, and stigma [7,37]. Sevenoaks et al. [38] outlined the need for adequate intervention strategies to focus on the provision of resources and psycho-social support for caregivers of ALHIV to improve their mental health and wellbeing. Evidence on the use of cash transfers plus behavioural change communication interventions remains scarce and inconclusive [39]. However, using low-cost mHealth alternatives to traditional behavioural change communication, namely, a nudge SMS package as in the trial, has been shown to result in positive outcomes for improved maternal health behaviour and outcomes associated with vaccinations in previous studies [39]. A growing body of evidence suggests the impact of behavioural economic interventions such as cash transfers or nudge-informed SMS messages for improving HIV care outcomes [40–42] or COVID-19 preventative measures. The current trial adds to the body of knowledge related to use of a multipronged approach to improve wellbeing outcomes for caregivers of ALHIV.

Few studies have examined the burden that caregivers of ALHIV encounter, particularly in SSA [43,44]. This vulnerable group of adolescents encounter unique challenges which predispose their caregivers to a significant burden that stems from prolonged caregiving in the context of the unique stressors associated with caring for ALHIV [43]. Caregivers in the intervention arm had a 1.28 unit (p = 0.020) decrease in their caregiver burden scores at four months compared to the control arm. Our results concur with an earlier Ugandan trial which found that a cash transfer plus family-based economic and psycho-social support programme was associated with significant reductions in caregiver distress (β = −4.9, 95% CI = −8.5, −1.4). [45]. The significant decrease in caregiver burden scores in the CWEL intervention arm could be attributed to the cash transfer that allowed caregivers to purchase basic food and school items which resulted in alleviating day-to-day stresses such as having to borrow money for these basic needs as evidenced in the qualitative data. The decrease in caregiver burden scores could also be a result of the content of the nudge SMS messages, where caregivers in the IDIs shared stories of the messages being a source of emotional and spiritual support when they needed to cope with their high burdened caregiving responsibilities during the COVID-19 pandemic. Previous reviews support this finding, where there has been evidence of positive effects due to psycho-social-based interventions (such as information sharing, and telephone support) on caregiver burden within 3 months [46]. Although our study design did not permit investigating the effect changes by the type of behavioural nudge message, a recent South African study found that loss aversion framing messages increased timely ART clinic visits [17]. A larger trial is needed to confirm these findings and examine the effects of a multipronged approach that uses economic incentives combined with economic empowerment or counselling intervention components.

Drawing on our survey and qualitative data, several factors may have limited us from detecting significant intervention effects for the outcomes of the trial. Firstly, the short duration of the intervention (three months) and the monthly cash transfer value (R350 ZAR, $21 USD) being below the national household poverty threshold (R945 ZAR, $58 USD) and monthly wage (R3864 ZAR, $236 USD) in 2022 may have been inadequate to impact wellbeing and depressive symptoms in the context of high levels of unemployment, coupled with the loss of household breadwinners and increased food insecurity during the COVID-19 pandemic. Secondly, our qualitative data revealed that participants may have had low levels of self-efficacy as participants in the intervention arm reported they did not access the free psycho-social support service highlighted in the nudge SMS package. Likely, the high prevalence of HIV-related stigma and other intersecting stigmas such as mental health and poverty in this context may impede linkage to mental health care, as evidenced in previous studies [47]. The findings suggest that to ensure significant improvements in wellbeing and mental health outcomes, cash transfer amounts should be informed by household poverty threshold values in our setting with more active referrals to support improved linkage to mental health care.

The pilot trial also found promising results for feasibility, acceptability and cost. Recruitment and retention were favourable, suggesting that trials among caregivers of ALHIV are feasible in SSA. Whilst pre-post differences in the outcome scores by arm were relatively modest, the use of cash transfers plus nudge SMS messages for caregivers does warrant further investigation given the high prevalence of poor psychological wellbeing, depressive symptoms and caregiver burden in our sample. Future studies should include a sub-sample of caregivers with clinically diagnosed depression to assess effects and consider exploring caregivers of adolescents with other chronic conditions. Our results support the feasibility of mobile technology for the delivery of cash transfers or psycho-social programming for caregivers during or as part of post-pandemic recovery efforts as our intervention was associated with high uptake with most participants being able to access the electronic ATM during the pandemic, and there were no safety or serious adverse events reported. South Africa lacks local cost-effectiveness thresholds and therefore we compared our estimated ICERs to the country’s GDP. Whilst this approach has major limitations [7,34], our reported ICERs (USD $1,080–1,100) were below South Africa’s GDP per capita which suggests that the intervention is cost-effective. Furthermore, these ICERs were comparable to that reported in a trial of a family-based economic empowerment intervention in Uganda [48]. The average cost per participant of the intervention (USD $259–271) is also comparable to that documented in other caregiver mental health [48] or adolescent HIV intervention [49] evaluations in the region. At present, social programmes within government HIV clinics are scarce limiting the ability to absorb some of the key intervention cost drivers (building, furniture and equipment, utilities). The total cost of the intervention could be reduced if integrated with existing government social policy initiatives outside the health sector (e.g., the Social Relief of Distress grant) as overheads would be primarily covered by this broader programme. To assess the affordability and sustainability of the intervention, future implementation science studies should assess various payment structures of the financial incentives, integration of the intervention in existing primary mental healthcare or other social programmes, coupling the intervention with economic empowerment programmes focused on caregiving and income generation activities, as well as directing part of the cash transfer towards improving adolescent outcomes (e.g., HIV testing) to ensure broader benefit to the familial network. More importantly, social impact bonds for health and national health insurance policies are being considered in the region, which might be a valid direction for future research in an assessment of whether private funders, insurance companies, government or public payers will invest in cash transfers to improve wellbeing and mental health outcomes. Lastly, the ethical considerations of nudges (i.e., autonomy, and long-term adverse effects) require further investigation in this context.

The strengths of this trial include the co-development of our intervention with caregivers of ALHIV from the study setting; added to adequate retention in both arms at follow-up; and the incorporation of a qualitative and costing study alongside the trial. We acknowledge several limitations of this pilot trial, which include: 1) the psychometric properties of the scales used to measure our primary and secondary outcomes among caregivers in South Africa are unknown. However, we drew on these scales as these have been validated in other adult populations in South Africa and caregiver groups in Europe. Furthermore, we translated the scales into isiZulu and conducted brief cognitive interviews to assess understanding; 2) whilst previous studies have demonstrated the benefits of cash transfers on improving health outcomes, failure to include a control arm that received a cash transfer created a resource gap in this trial. Hence, participants in the control arm may have been less likely to alleviate financial stresses compared to the intervention arm; 3) due to budget restrictions, the trial did not include an arm that was provided only one component of the intervention (cash transfer or nudge SMS package only). This prevented us from assessing the individual effects of the intervention components; 3) due to funding constraints our sample size and intervention delivery period was restricted. However, this was an initial proof of concept pilot trial. A future study that is powered to assess all outcome effects with longer timespan is needed; 4) selection bias is likely to be present as there were significantly more participants with depressive symptoms in the intervention versus control arm at baseline; with higher lost to follow-up reported in the control arm at endline likely due to the lower levels of study engagement with this arm 5) due to social desirability bias, not all participants would have been forthcoming about household income and receipt of the government’s COVID-19 relief efforts, making it difficult to delineate the effect associated with the financial incentive; 6) the duration and frequency may have been inadequate to assist with daily behaviour changes towards positive mental health coping. This theory would be consistent with behavioural economics insights that the frequency and immediacy of costs and benefits can have strong influences on behaviour [50]; 7) our results may only be generalisable to caregivers from peri-urban African settings; 8) it is likely there was social mixing among trial participants in the community and thus a decrease in flourishing levels observed in the control arm may have been linked to intervention arm participants disclosing receipt of the cash transfer and its positive effects on their wellbeing. Future studies should minimise contamination and assess knowledge levels of trial arms, finally, 9) this was a “real world” pragmatic trial, where intervention participants who received the cash transfer could have been able to commute to services or call members in their support networks. Whilst we did not find any evidence of this in our qualitative interviews, the absence of an attention control group means we cannot exclude the possibility that this additional contact was responsible for observed effects favouring the intervention arm.

## Conclusion

In this pilot RCT, we investigated a behavioural economic package (cash transfer plus nudge SMS) for improving caregiver wellbeing and mental health during the COVID-19 pandemic. Whilst the intervention did not have a significant effect on wellbeing and depressive symptoms, point estimates indicate a shift in scores in favour of the intervention. Of note, the intervention was associated with a significant decrease in caregiver burden suggesting that this approach could help alleviate the daily stressors of caregiving during a pandemic. Furthermore, we showed that delivery of a cash transfer plus nudge SMS package is feasible with regards to participant acceptability and cost for resource limited settings. Future studies should aim to confirm these findings through a larger trial and enhancing the intervention by increasing the cash transfer amount to the household poverty threshold value, extending its duration, and incorporating elements of HIV-related intersectional stigma and economic empowerment.

## Supporting information

S1 Fig CweL trial design. (TIF)

S1 Table Nudge Messages. (DOCX)

S2 Table Description of cost resource collection. (DOCX)

S3 Table Definition of outcomes. (DOCX)

S4 Table Sample size calculations. (DOCX)

S5 Table Baseline characteristics - retained versus not-retained. (DOCX)

S6 Table Primary and secondary outcome scores. (DOCX)

S7 Table Changes in primary and secondary outcomes at endline. (DOCX)

S8 Table ICER calculations. (DOCX)
