# Supplementary material for: Using a cash transfer plus SMS nudge package to improve the wellbeing among caregivers of adolescents living with HIV during the COVID-19 epidemic in South Africa: A pilot randomised controlled trial
Source: PLOS Glob Public Health. 2025 May 16;5(5):e0003799. doi: 10.1371/journal.pgph.0003799 (PMC12083824; doi:10.1371/journal.pgph.0003799)
Supplement: S1 Table — (DOCX) [file pgph.0003799.s002.docx]

# S1 Table: Nudge Messages

**Table 1: Nudge-based mobile SMS delivered to intervention participants over a 3-month period**

| **Message** | **Behavioural Economic principle** | **Excerpts from mobile message** |
| --- | --- | --- |
| **1** | **Loss aversion** | *“If you need to speak to someone you can call the South African Depression and Anxiety Group at no cost to you (Number:)”* |
| **2** | **Aspiration framing** | *“Taking care of your own needs is important so you can be there to watch your child grow”* |
| **3** | **Altruism** | *“Taking care of your child and family’s needs are important”* |
