# Supplementary material for: Using a cash transfer plus SMS nudge package to improve the wellbeing among caregivers of adolescents living with HIV during the COVID-19 epidemic in South Africa: A pilot randomised controlled trial
Source: PLOS Glob Public Health. 2025 May 16;5(5):e0003799. doi: 10.1371/journal.pgph.0003799 (PMC12083824; doi:10.1371/journal.pgph.0003799)
Supplement: S2 Table — (DOCX) [file pgph.0003799.s003.docx]

# S2 Table: Description of cost resource collection

| **Table 1: Detailed Description of Cost Resource Collection: Methods** | | | |
| --- | --- | --- | --- |
|  | **Description** | |  |
| **Cost Category** | **Intervention** | **Control** | **Primary method of data collection and data source** |
| ***Fixed costs (Capital)*** |  |  |  |
| Building | Applies to the field office building: rooms where workshops, development trainings, delivery, monitoring, and reporting occurred | Applies to the field office building: rooms where development, trainings, delivery, monitoring, and reporting occurred | - Direct measurement of the room size - Protect roster-Days used by project |
| Vehicle | 5 seater passenger car used for the purpose of the intervention development- transportation of workshop participants |  | - Time use of vehicle by project- log book - Financial records- book value of car - Annuitisation |
| Furniture & equipment | Furniture and equipment used by staff and participants in workshops, trainings, delivery (e.g. laptops, mobile phones, audio-recorders, speakers, tables, chairs) | Furniture and equipment used by staff in trainings, delivery  (e.g. laptops, mobile phones, tables, chairs) | - Time use of assets by project - Project financial records |
| Development costs | Applies to SOP and database development and trainings, and set-up of the cash transfer system (staff time, printing, stationery) | This applies to manual and database development, and trainings  (staff time, printing, stationery) | - Staff timesheets - Financial records |
|  |  |  |  |
| ***Variable costs (recurrent)*** |  |  |  |
| Operational staff | Staff involved in the intervention delivery: monitoring, refresher training, addressing queries  (project manager, co-ordinator, fieldworkers) | Staff involved in the control delivery: monitoring, refresher training, addressing queries  (project manager, co-ordinator, fieldworkers) | - Time sheets - Proportion of their time at work that is spent on the project |
| Supplies | Applies to all supplies used in the intervention delivery  (cash incentives, stationery, printing, airtime, and data for mobile phones) | Applies to all supplies used in the control delivery  (stationery, printing, airtime for mobile phones) | - Direct measure - Project financial records |
| Utilities | Applies to building operating and maintenance (cleaner, security, electricity and water) and vehicle operation (fuel) | Applies to building operating and maintenance (cleaner, security, electricity and water) and vehicle operation (fuel) | - Proportion of time building was used (project roster) - Proportion of time vehicle was used (kilometres covered)- log book |
| Caregiver out of pocket payment | Direct cost participants incurred because of accessing the intervention (i.e.transport costs to the nearest ATM to withdraw their cash and attend the clinic) | Direct cost participants incurred because of accessing the control (i.e. transport costs attend the clinic) | - Cash transfer banking report - Project database- follow-up interviews |
