# Supplementary material for: Using a cash transfer plus SMS nudge package to improve the wellbeing among caregivers of adolescents living with HIV during the COVID-19 epidemic in South Africa: A pilot randomised controlled trial
Source: PLOS Glob Public Health. 2025 May 16;5(5):e0003799. doi: 10.1371/journal.pgph.0003799 (PMC12083824; doi:10.1371/journal.pgph.0003799)
Supplement: S3 Table — (DOCX) [file pgph.0003799.s004.docx]

# S3 Table: Definition of outcomes

**Table 1: Description of scoring and categorisation of primary and secondary outcome measures**

| **Variable** | **Survey questions** | **Survey response options** | **Recoding of survey options** | **Scoring or categorisation** |
| --- | --- | --- | --- | --- |
| **Primary outcomes** |  |  |  |  |
| Psychological wellbeing  **(MHC-SF)** | *During the past month how often did you feel ...*  1. happy  2. interested in life  3. satisfied  4. that you had something important to contribute to society e.g. assisting with community events  5. that you belonged to a community like a social group, or your neighbourhood)  6. that our society is becoming a better place for people like you  7. that people are basically good  8. that the way our society works makes sense to you  9. that you liked most parts of your personality  10. good at managing the responsibilities of your daily life  11. that you had warm and trusting relationships with others  12. that you had experiences that challenged you to grow and become a better person  13. confident to think or express your own ideas and opinions  14. that your life has a sense of direction or meaning to it | 1 = Never  2 = Once or twice  3 = About once a week  4 = About 2 to 3 times a week  5 = Almost every day  6 = Every day | 0 = Never  1 = Once or twice  2 = About once a week  3 = About 2 to 3 times a week  4 = Almost every day  5 = Every day | **Continuous scoring:** Summation of the 14-item scale, total score range 0-70  **Categorical scoring:**  **0 = Flourishing**  If ≥1 of 3 hedonic wellbeing symptoms (items 1-3) responses 5-6 and ≥6 of 11 eudaimonic signs (social well-being (items 4-8) and psychological wellbeing (items 9-14)) responses 5-6.  **3 = Languishing**  If ≥1 of 3 hedonic wellbeing symptoms (items 1-3) responses 1-2 and ≥6 of 11 eudaimonic signs (social well-being (items 4-8) and psychological wellbeing (items 9-14)) responses 1-2.  **2 = Moderately mentally health**  If neither “languishing” nor “flourishing”.  [**https://peplab.web.unc.edu/wp-content/uploads/sites/18901/2018/11/MHC-SFoverview.pdf**](https://peplab.web.unc.edu/wp-content/uploads/sites/18901/2018/11/MHC-SFoverview.pdf) |
| Subjective wellbeing | On a scale of 1-10, how happy do you feel at the moment? | Scale 1-10 | NA | **Continuous scoring:** Total score ranging 1-10  **Categorical scoring:**  0 = No (total score < 5)  1 = Yes (total score >= 5) |
| **Secondary outcomes** |  |  |  |  |
| Depressive symptoms (CES-D-10) | *During the Past Week…*  1. I was bothered by things that usually don’t bother me.  2. I had trouble keeping my mind on what I was doing.  3. I felt depressed  4. I felt that everything I did was an eff ort.  5. I felt hopeful about the future.  6. I felt fearful.  7. My sleep was restless.  8. I was happy.  9. I felt lonely.  10. I could not "get going." | 1 = Rarely or none of the time (less than 1day) 2 = Some or a little of the time (1-2 days) 3 = Occasionally or a moderate amount of time (3-4 days) 4 = Most or all of the time (5-7 days) | **Items 5 and 8 recoded to:** 0 = Most or all of the time (5-7 days) 1 = Occasionally or a moderate amount of time (3-4 days) 2 = Some or a little of the time (1-2 days) 3 = Rarely or none of the time (less than 1day) **Items 1-4, 6-7, and 9-10 recoded to:** 0 = Rarely or none of the time (less than 1day) 1 = Some or a little of the time (1-2 days) 2 = Occasionally or a moderate amount of time (3-4 days) 3 = Most or all of the time (5-7 days) | **Continuous scoring:** Summation of the 10-item scale, total score range 0-30  **Categorical scoring:**  0 = No (total score <12)  1 = Yes (total score >=12)  ([1](#_ENREF_1)) |
| Caregiver burden | Please only select one option per description: 'no', 'some' or 'a lot of'  1. I have _________ fulfilment from carrying out my care tasks.  2. I have _________ relational problems with the care receiver (e.g., she/he is very demanding, or he/she behaves differently; we have communication problems).  3. I have _________ problems with my own mental health (e.g., stress, fear, gloominess, depression, concern about the future).  4. I have _________ problems combining my care tasks with my daily activities (e.g., household activities, work, study, family and leisure activities).  5. I have _________ financial problems because of my care tasks.  6. I have _________ support with carrying out my care tasks, when I need it (e.g., from family, friends, neighbours, acquaintances).  7. I have _________ problems with my own physical health (e.g., more often sick, tiredness, physical stress). | 1 = No  2 = Some  3 = A lot | **Responses to items 1 and 6 recoded to:**  0 = A lot 1 = Some 2 = No   **Responses to items 2-5 and 7 recoded to:**  0 = No 1 = Some 2 = A lot | **Continuous scoring:** Responses weighted using Polish Tariff  ([2](#_ENREF_2), [3](#_ENREF_3)) **Categorical scoring:**  0 = No (Total score <median score) 1 = Yes (Total score >= median score) |

1. Baron EC, Davies T, Lund C. Validation of the 10-item Centre for Epidemiological Studies Depression Scale (CES-D-10) in Zulu, Xhosa and Afrikaans populations in South Africa. BMC Psychiatry. 2017;17:1-14.

2. Brouwer WBF, van Exel NJA, van Gorp B, Redekop WK. The CarerQol instrument: A new instrument to measure care-related quality of life of informal caregivers for use in economic evaluations. Quality of Life Research. 2006;15(6):1005-21.

3. Baji P, Farkas M, Golicki D, Prevolnik Rupel V, Hoefman R, Brouwer WBF, et al. Development of Population Tariffs for the CarerQol Instrument for Hungary, Poland and Slovenia: A Discrete Choice Experiment Study to Measure the Burden of Informal Caregiving. PharmacoEconomics. 2020;38(6):633-43.
