# Supplementary material for: Using a cash transfer plus SMS nudge package to improve the wellbeing among caregivers of adolescents living with HIV during the COVID-19 epidemic in South Africa: A pilot randomised controlled trial
Source: PLOS Glob Public Health. 2025 May 16;5(5):e0003799. doi: 10.1371/journal.pgph.0003799 (PMC12083824; doi:10.1371/journal.pgph.0003799)
Supplement: S4 Table — (DOCX) [file pgph.0003799.s005.docx]

# S4 Table: Sample size calculations

| Table 1: Estimated sample size for repeated-measures ANOVA  F test for between subjects  Ho: delta = 0 versus Ha: delta != 0 | | | | | | | | |  |  |
| --- | --- | --- | --- | --- | --- | --- | --- | --- | --- | --- |
| alpha | **power** | **N** | **N_per_group** | **delta** | **N_g** | **N_rep** | **Var_b** | **Var_be** | **Var_e** | **corr** |
| .05 | .8 | 24 | 12 | .6217 | 2 | 2 | 2.3 | 5.95 | 7 | .7 |
| .05 | .8 | 22 | 11 | .6351 | 2 | 2 | 2.4 | 5.95 | 7 | .7 |
| .05 | .8 | 22 | 11 | .6482 | 2 | 2 | 2.5 | 5.95 | 7 | .7 |
| .05 | .8 | 22 | 11 | .661 | 2 | 2 | 2.6 | 5.95 | 7 | .7 |
| .05 | .8 | 26 | 13 | .5816 | 2 | 2 | 2.3 | 6.8 | 8 | .7 |
| .05 | .8 | 26 | 13 | .5941 | 2 | 2 | 2.4 | 6.8 | 8 | .7 |
| .05 | .8 | 24 | 12 | .6063 | 2 | 2 | 2.5 | 6.8 | 8 | .7 |
| .05 | .8 | 24 | 12 | .6183 | 2 | 2 | 2.6 | 6.8 | 8 | .7 |
| .05 | .8 | 30 | 15 | .5483 | 2 | 2 | 2.3 | 7.65 | 9 | .7 |
| .05 | .8 | 28 | 14 | .5601 | 2 | 2 | 2.4 | 7.65 | 9 | .7 |
| .05 | .8 | 28 | 14 | .5717 | 2 | 2 | 2.5 | 7.65 | 9 | .7 |

• power repeated, varbetween(2.3 2.4 2.5 2.6) ngroups(2) nrepeated(2) corr(0.7) varerror(7 8 9 10)
