# Supplementary material for: Using a cash transfer plus SMS nudge package to improve the wellbeing among caregivers of adolescents living with HIV during the COVID-19 epidemic in South Africa: A pilot randomised controlled trial
Source: PLOS Glob Public Health. 2025 May 16;5(5):e0003799. doi: 10.1371/journal.pgph.0003799 (PMC12083824; doi:10.1371/journal.pgph.0003799)
Supplement: S5 Table — (DOCX) [file pgph.0003799.s006.docx]

# S5 Table: Baseline characteristics – retained versus not-retained

**Table a: Baseline characteristics of those retained at follow-up**

| **Characteristic** |  | **Control (n=37)** | **Intervention (n=48)** | **p-value** |
| --- | --- | --- | --- | --- |
| Caregiver age (years) mean (IQR) years |  | 40 (33-47) | 43 (33-48) | 0.690 |
| Age categories (years) | <=60 | 35 (95) | 46 (96) | 0.790 |
|  | >60 | 2 (5) | 2 (4) |  |
| Gender | Male | 6 (16) | 6 (12) | 0.630 |
|  | Female | 31 (84) | 42 (88) |  |
| Employment status | Unemployed | 21 (57) | 30 (62) | 0.590 |
|  | Employed | 16 (43) | 18 (38) |  |
| Married | No | 14 (38) | 19 (40) | 0.870 |
|  | Yes | 23 (62) | 29 (60) |  |
| Recipient of any social grant | No | 7 (19) | 4 (8) | 0.150 |
|  | Yes | 30 (81) | 44 (92) |  |
| HIV-status | HIV-positive | 37 (100) | 47 (100) |  |
| Household food insecurity (FIES) | Mild | 17 (46) | 12 (25) | 0.011 |
|  | Moderate | 9 (24) | 6 (12) |  |
|  | Severe | 11 (30) | 30 (62) |  |
| Psychological wellbeing (MHC) | Flourishing | 24 (65) | 19 (40) | 0.041 |
|  | Moderately Mentally Healthy to languishing | 13 (35) | 29 (60) |  |
| Subjective wellbeing-(CarerQol-VAS) | Mean happiness score | 7 (5-9) | 5 (3-7) | 0.005 |
| Depressive symptoms  (CESD-10) | No | 16 (43) | 11 (23) | 0.046 |
|  | Yes | 21 (57) | 37 (77) |  |
| Caregiver burden (CESD-10) | No | 18 (49) | 18 (38) | 0.300 |
|  | Yes | 19 (51) | 30 (62) |  |

**Table b: Baseline characteristics of those not retained^ at endline**

| **Characteristic** |  | **Control (n=13)** | **Intervention**  **(n=2)** | **p-value** |
| --- | --- | --- | --- | --- |
| Caregiver age (years), mean (IQR) |  | 48 (39-53) | 44 (28-60) | 0.730 |
| Age categories years | <60 | 10 (77) | 2 (100) | 0.450 |
|  | >60 | 3 (23) | 0 (0) |  |
| Gender | Male | 2 (15) | 0 (0) | 0.550 |
|  | Female | 11 (85) | 2 (100) |  |
| Employment status | Employed | 8 (62) | 0 (0) | 0.100 |
|  | Unemployed | 5 (38) | 2 (100) |  |
| Married | No | 7 (54) | 1 (50) | 0.920 |
|  | Yes | 6 (46) | 1 (50) |  |
| Recipient of social grant | No | 1 (8) | 0 (0) | 0.680 |
|  | Yes | 12 (92) | 2 (100) |  |
| HIV-status | HIV-positive | 13 (100) | 2 (100) |  |
| Household food insecurity (FIES) | Mild | 1 (8) | 1 (50) | 0.240 |
|  | Moderate | 3 (23) | 0 (0) |  |
|  | Severe | 9 (69) | 1 (50) |  |
| Psychological wellbeing (MHC-SF) | Flourishing | 4 (31) | 2 (100) | 0.063 |
|  | Moderately Mentally Healthy to languishing | 9 (69) | 0 (0) |  |
| Subjective wellbeing (CarerQol-VAS) | Mean happiness score | 4 (2-6) | 10 (10-10) | 0.038 |
| Depressive symptoms (CESD-10) | No | 2 (15) | 1 (50) | 0.250 |
|  | Yes | 11 (85) | 1 (50) |  |
| Caregiver burden | No | 4 (31) | 1 (50) | 0.590 |
|  | Yes | 9 (69) | 1 (50) |  |

^= lost to follow-up or deceased
