# Supplementary material for: Using a cash transfer plus SMS nudge package to improve the wellbeing among caregivers of adolescents living with HIV during the COVID-19 epidemic in South Africa: A pilot randomised controlled trial
Source: PLOS Glob Public Health. 2025 May 16;5(5):e0003799. doi: 10.1371/journal.pgph.0003799 (PMC12083824; doi:10.1371/journal.pgph.0003799)
Supplement: S6 Table — (DOCX) [file pgph.0003799.s007.docx]

# S6 Table: Primary and secondary outcome scores

| Table 1: Primary and secondary outcome scores at baseline versus endline | | | | | | |
| --- | --- | --- | --- | --- | --- | --- |
|  | **Baseline**  **Mean score (SD)** | |  | **Endline**  **Mean score (SD)** | |  |
|  | **Intervention** | **Control** | **Difference** | **Intervention** | **Control** | **Difference** |
| Primary outcomes: | | | | | | |
| Psychological wellbeing  (MHC- SF) | 45.82  (15.89) | 47.84 (14.70) | -2.02 | 46.43  (14.82) | 48.05 (13.46) | -1.62 |
| Subjective wellbeing  (Carer QoL VAS) | 5.26  (2.87) | 6.22 (2.53) | -0.96 | 5.55  (1.99) | 6.22  (1.89) | -0.66 |
| Secondary outcome: | | | | | | |
| Depressive symptoms  (CESD-10) | 16.10  (5.32) | 13.76 (5.34) | 2.34 | 14.85  (4.29) | 12.27  (4.68) | 2.58 |
| Caregiver burden  (Carer QoL) | 7.58  (3.08) | 6.20 (2.89) | 1.38 | 7.15  (1.99) | 6.46  (2.73) | 0.67 |
| SD= standard deviation, MHC-SF= Mental Health Continuum Short Form; Carer QoL VAS= Caregiver Quality of Life- Visual Analogue Scale; CESD-10= Center for Epidemiologic Studies Depression Scale- 10 items; Carer QoL= Caregiver Quality of Life Scale | | | | | | |
