# Supplementary material for: Using a cash transfer plus SMS nudge package to improve the wellbeing among caregivers of adolescents living with HIV during the COVID-19 epidemic in South Africa: A pilot randomised controlled trial
Source: PLOS Glob Public Health. 2025 May 16;5(5):e0003799. doi: 10.1371/journal.pgph.0003799 (PMC12083824; doi:10.1371/journal.pgph.0003799)
Supplement: S7 Table — (DOCX) [file pgph.0003799.s008.docx]

# S7 Table: Changes in primary and secondary outcomes at endline

| Table 1: Assessing changes in primary and secondary outcomes at endline (M4) (full sample) using linear and log-binomial regression models | | | | | | |
| --- | --- | --- | --- | --- | --- | --- |
| Outcome | **β (95% CI)** | | **Analysis** | | | |
| Continuous* | **Intervention** | **Control** | **Unadjusted**  **Mean difference (95% CI)** | **p-value** | **Adjusted^**  **Mean difference**  **β (95% CI)** | **p-value** |
| Primary outcome: |  |  |  |  |  |  |
| Psychological wellbeing (MHC- SF)* | 1.36 (-2.99-5.71) | -1.27 (-5.36 to 2.82) | 3.14 (-3.09 to 9.37) | 0.319 | 2.93 (-3.41 to 9.28) | 0.361 |
| Subjective wellbeing  (Carer QoL VAS)* | 0.51 (-0.39-1.40) | -0.54 (-1.30 to 0.22) | 1.05 (-0.15 to 2.25) | 0.085 | 0.95 (-0.26 to 2.15) | 0.123 |
| Secondary outcome: |  |  |  |  |  |  |
| Depressive symptoms  (CESD-10) | -1.38 (-2.75-0.001) | -0.05 (-1.53 to 1.42) | -1.32 (-3.3 to -0.68) | 0.193 | -1.40 (-3.44 to 0.63) | 0.173 |
| Caregiver burden  (Carer QoL) | -0.47 (-1.20-0.27) | 0.81 (0.03 to 1.60) | -1.28 (-2.35 to -0.21) | 0.020 | -1.21 (-2.30 to -0.13) | 0.029 |
| Binary** | **n/N (%)** | **n/N (%)** | **Unadjusted**  **Risk Ratio (95% CI)** | **p-value** | **Adjusted^**  **Risk Ratio (95% CI)** | **p-value** |
| Primary outcome: |  |  |  |  |  |  |
| Psychological wellbeing (MHC- SF) |  |  |  |  |  |  |
| Flourishing | 39/50 (78.0) | 44/50 (88.0) | reference |  | reference |  |
| Languishing/Moderately mentally healthy | 11/50 (22.0) | 6/50 (12.0) | 0.55 (0.22-1.36) | 0.194 | 0.75 (0.32-1.72) | 0.497 |
| Secondary outcome: |  |  |  |  |  |  |
| Depressive symptoms  (CESD-10) |  |  |  |  |  |  |
| Yes | 8/50 (16.0) | 4/50 (8.0) | 0.5 (0.16-1.55) | 0.231 | 1.00 (0.40-2.53) | 0.992 |
| No | 42/50 (84.0) | 46/50 (92.0) | reference |  | reference |  |
| Caregiver burden  (Carer QoL) |  |  |  |  |  |  |
| Yes | 11/50 (22.0) | 8/50 (16.0) | 0.73 (0.32-1.65) | 0.448 | 0.87 (0.44-1.73) | 0.695 |
| No | 39/50 (78.0) | 42/50 (84.0) | reference |  | reference |  |
| *= linear regression; **= log-binomial regression, ^= adjusted for age and sex at baseline; Abbreviations: MHC= Mental Health Continuum Short Form; Carer QoL VAS= Caregiver Quality of Life- Visual Analogue Scale; CESD-10= Center for Epidemiologic Studies Depression Scale- 10 items; Carer QoL= Caregiver Quality of Life Scale | | | | | | |
