# Supplementary material for: Using a cash transfer plus SMS nudge package to improve the wellbeing among caregivers of adolescents living with HIV during the COVID-19 epidemic in South Africa: A pilot randomised controlled trial
Source: PLOS Glob Public Health. 2025 May 16;5(5):e0003799. doi: 10.1371/journal.pgph.0003799 (PMC12083824; doi:10.1371/journal.pgph.0003799)
Supplement: S8 Table — (DOCX) [file pgph.0003799.s009.docx]

# S8 Table: ICER Calculations

**Table 1: Incremental Cost Effectiveness Ratio (ICER)- provider perspective**

|  | **Difference** | **Intervention** | **Control** |
| --- | --- | --- | --- |
| 1. **Average cost per participant (Total provider cost/n=50)** |  | $259 | $43 |
| 1. **Difference in average cost per participant** | $216 |  |  |
| 1. **Proportion of participants with an increase in psychological wellbeing score** |  | 0.46 | 0.26 |
| 1. **Difference in proportion of participants with increase in psychological wellbeing score** | 0.2 |  |  |
| **ICER (b/d)** | ($216/0.2) = $1,080 |  |  |

**Table 2: Incremental Cost Effectiveness Ratio (ICER)- societal perspective**

|  | **Difference** | **Intervention** | **Control** |
| --- | --- | --- | --- |
| 1. **Average cost per participant (Total societal cost/n=50)** |  | $271 | $49 |
| 1. **Difference in average cost per participant** | $222 |  |  |
| 1. **Proportion of participants with an increase in psychological wellbeing score** |  | 0.46 | 0.26 |
| 1. **Difference in proportion of participants with increase in psychological wellbeing score** | 0.2 |  |  |
| **ICER (b/d)** | ($222/0.2) = $1,100 |  |  |
